# Un lymphome T/NK extra-ganglionnaire de type nasal se présentant comme une cellulite orbitaire

**DOI:** 10.11604/pamj.2018.31.8.16221

**Published:** 2018-09-04

**Authors:** Ghizlan EL Amri, Mohamed Zalagh, Fouad Benariba

**Affiliations:** 1 Service d’ORL et de Chirurgie Cervico-faciale, Hôpital Militaire d’Instruction Mohamed V, Faculté de Médecine, Université Mohamed V, Rabat, Maroc

**Keywords:** Lymphome T/Nk extra-ganglionnaire, cellulite orbitaire, sinusite, Extra-nodal NK/T-cell lymphoma, orbital cellulitis, sinusitis

## Abstract

Le lymphome extra-ganglionnaire T/NK de type nasal est une forme rare des lymphomes malins non hodgkinien. Elle pose un problème diagnostique à cause des symptômes peu spécifiques. Nous rapportons un cas de lymphome T/NK simulant une cellulite orbitaire. Il s'agit d'une patiente de 46 ans, suivie pour maladie de Behçet. Admise dans un tableau clinique de cellulite orbitaire. Le scanner du massif facial montrait une pansinusite avec infiltration orbitaire. En l'absence d'amélioration sous traitement antibiotique. Un traitement chirurgical avec biopsie a été réalisé. L'examen histologique a mis en évidence des signes de vascularite inflammatoire et la patiente a été mise sous corticothérapie avec amélioration clinique. Devant la récidive la patiente a bénéficié d'une reprise chirurgicale avec des biopsies de l'ethmoïde qui ont mis en évidence un lymphome T/NK de type nasal. Le traitement a consisté en une radio-chimiothérapie et la patiente est décédée dans les deux mois. Les lymphomes T/NK sont agressifs, ils touchent essentiellement les cavités naso-sinusiennes. Ils sont responsables d'une angio-destruction et de la nécrose qui rendent les symptômes peu spécifiques et les biopsies souvent négatives, posant un problème de diagnostic différentiel. Le traitement repose sur la radiothérapie et la chimiothérapie et le pronostic reste réservé.

## Abstract (en)

Extra-nodal NK/T-cell lymphoma, nasal type, is a rare form of malignant non-Hodgkin’s lymphomas. It poses a diagnostic problem due to nonspecific symptoms. We report a case of NK/T-cell lymphoma simulating orbital cellulitis. The study involved a 46-year old female patient followed up for Behçet’s disease. The patient was admitted with orbital cellulitis. CT scan of the midface showed pansinusitis with orbital infiltration.The patient had no improvement under antibiotic therapy. Surgical treatment with biopsy was performed. Histological examination showed inflammatory vasculitis and the patient underwent corticosteroid therapy with clinical improvement. The patient had recurrence requiring reoperation with ethmoid biopsies whose histological examination showed NK/T-cell lymphoma, nasal type. Treatment was based on radiochemotherapy and the patient died after two months. NK/T-cell lymphomas are aggressive, they primarily affect the nasal cavities and sinus. They cause angiodestruction and necrosis, resulting in unspecific symptoms. Biopsies are often negative, posing a problem of differential diagnosis. Treatment is based on radiotherapy and chemotherapy. Prognosis is reserved.

## Introduction

Le lymphome extra-ganglionnaire T/NK (Natural killer) (LTNK) de type nasal est une forme rare des lymphomes malins non hodgkinien, représentant 1,4% de tous les lymphomes [1]. Il est hautement invasif, et siège avec prédilection au niveau des cavités nasales, du nasopharynx et des sinus para-nasaux. Cette entité pose un problème diagnostique à cause des symptômes peu spécifiques. C'est le cas d'une localisation ethmoïdo-orbitaire révélée par une cellulite orbitaire.

## Patient et observation

**Figure 1:**
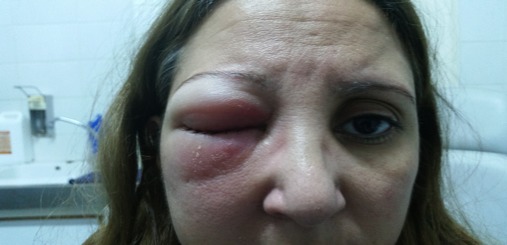
Tuméfaction inflammatoire des paupières étendue à la joue

**Figure 2:**
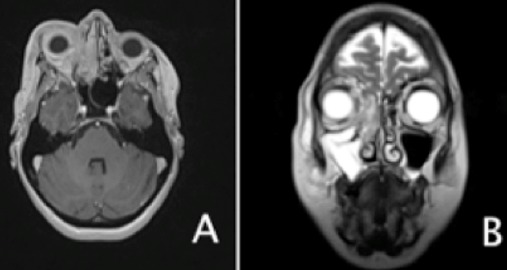
IRM en coupe axiale T1 avec injection de gadolinium (A) et en coupe coronale T2 (B) montrant un comblement tissulaire de l'ethmoide droit étendu à l'orbite homolatéral

**Figure 3:**
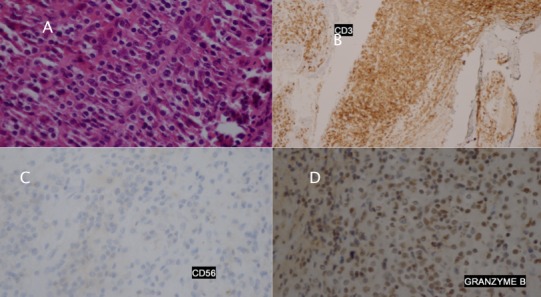
A) HES (100x) montrant l'infiltration par une dense population de cellules lymphomateuses; B) immunohistochimie montrant la positivité au CD3; C) immunohistochimie montrant la négativité des CD56; D) immunohistochimie montrant la positivité du Granzyme B

Il s'agit d'une patiente de 46 ans, suivie pour maladie de Behçet sans atteinte ophtalmologique. Elle a été admise dans un tableau clinique de cellulite orbitaire droite. L'examen clinique a trouvé une tuméfaction inflammatoire des paupières droites étendue à la joue homolatérale, un chémosis, une exophtalmie axile, une diminution de la motilité oculaire et de l'acuité visuelle (6/10 à droite, 9/10 à gauche). Cependant, la patiente ne présentait pas de signes rhinologiques. L'endoscopie nasale a visualisé du pus au niveau du méat moyen droit sans lésion tumorale. Il n'y avait pas d'adénopathie palpable, en particulier cervicale. La patiente était apyrétique et n'avait pas maigri (Figure 1). La C réactive protéine était à 16,3 mg/l, sans hyperleucocytose à l'hémogramme. L'examen microbiologique a révélé un Pseudomonas Aeroginosa. Le scanner et l'IRM du massif facial ont montré un comblement tissulaire de tous les sinus antérieurs droits et infiltrant la graisse orbitaire droite sans ostéolyse (Figure 2). Le diagnostic de cellulite orbitaire compliquant une pan-sinusite a été retenu. Et la patiente a été traitée par une bi-antibiothérapie adaptée à l'antibiogramme sans amélioration. Une méatotomie moyenne et une ethmoidectomie droites endoscopiques endonasales ont été réalisées. L'examen histologique avait conclu à une vascularite inflammatoire évoquant la maladie de Behçet. La patiente a été mise sous corticothérapie avec amélioration clinique. Cinq jours, après l'arrêt des corticoïdes, l'évolution a été marquée par la récidive de la symptomatologie et la patiente fut reprise chirurgicalement avec des biopsies larges et multiples de l'ethmoïde. L'examen histologique a montré des lymphocytes atypiques. Les marqueurs immunohistochimiques anti- CD3 et Granzyme B étaient positifs alors que les anti- CD20, CD5 et CD56 étaient négatifs et ces caractéristiques étaient compatibles avec un LTNK de type nasal à CD56 négatif (Figure 3). La biopsie ostéo-médullaire n'a pas montré d'envahissement. Le scanner thoraco-abdomino-pelvien et TEP scanner n'ont pas montré d'autres localisations. La patiente a été classée IE de la classification de Ann Arbor pour les lymphomes. Elle a bénéficié d'une radio-chimiothérapie, à base de cyclophosphamide, doxorubicine, vincristine, prednisolone (CHOP). La patiente est décédée dans les 2 mois au cours de la chimiothérapie.

## Discussion

Le LTNK de type nasal est une forme rare de lymphomes malins non hodgkinien, il dérive des cellules NK et des lymphocytes T cytotoxiques. Sa pathogénie est inconnue mais un lien avec le virus Epstein Barr est probable [2]. Il est fréquent en Asie et en Amérique centrale, où il représente 7 à 10% des lymphomes malins non hodgkiniens [1] . Les atteintes extra-ganglionnaires concernent le plus souvent le nez, le cavum et les sinus para-nasaux dans 70% des cas [2, 3]. La peau, le sein, le testicule et le tractus gastro-intestinal peuvent être atteints [3]. Les lymphomes NK/T sont localement agressifs. L'angio-destruction entraine de la nécrose qui peut être siège de surinfection. Les symptômes sont peu spécifiques et les biopsies sont négatives; ce qui pose un problème de diagnostic différentiel avec les rhino-sinusites, les mycoses et les maladies de systèmes dont la maladie de Behçet. Quelques rares cas de LTNK se présentant comme une rhinosinusite compliquée d'une cellulite orbitaire ont été décrits dans la littérature [4, 5]. Termote et al. ont décrit 3 cas similaires, traités par radio-chimiothérapies et décédés dans les 5 à 35 mois après le diagnostic [4]. Trois autres cas de LTNK avec atteinte orbitaire ont été rapportés par Charton et al. Ils ont été traités par antibiothérapie avant la biopsie qui a posé le diagnostic [5]. L'atteinte orbitaire est généralement le résultat d'une extension à partir de la cavité nasale et des sinus paranasaux avec ou sans lyse osseuse [3]. Cependant, Woog et al. ont rapporté que trois des huit cas de LTNK extra-ganglionnaire avaient une atteinte orbitaire isolée, annexielle et/ou oculaire [6]. Dans notre cas la patiente n'a pas rapporté de signes rhinologiques mais l'endoscopie nasale a objectivé une muqueuse nasale inflammatoire avec œdème et secrétions purulentes justifiant une antibiothérapie. La mise en évidence de signes de vascularite sur les premières biopsies a évoqué une manifestation rhino-sinusienne de la maladie de Behçet. En 2003, Martins et al. ont rapporté le premier cas d'une association entre une rhinosinusite destructive et le syndrome de Behçet chez un patient de 47 ans [7]. En outre, Alcântara LJ et al. ont rapporté un cas de rhinosinusite compliqué d'abcès périorbitaire qui serait lié à la vascularite nécrosante de la maladie de Behçet. La rhinosinusite est une manifestation potentielle de la maladie de Behçet que les praticiens doivent prendre en considération [8]. Cette notion a retardé le diagnostic de LTNK chez notre patiente. Le traitement dépend de l'extension et du stade de la maladie selon la classification de Ann Arbor. La maladie localisée est traitée avec une chimio-radiothérapie à visée curative, alors que la chimiothérapie est le pilier du traitement des LTNK diagnostiqués à un stade avancé [9]. Le pronostic du LTNK extra-ganglionnaire décrit dans la littérature reste variable; mais, généralement la maladie est rapidement progressive et le délai de survie est court à partir du moment du diagnostic. Il présente un taux de mortalité élevé et une faible réponse à la chimiothérapie et à la radiothérapie par rapport aux autres sous-types de lymphomes du cou et de la tête. La survie moyenne est de 12,5 mois après le diagnostic [10].

## Conclusion

Le LTNK est une tumeur invasive de mauvais pronostic à cause du retard diagnostic et du caractère destructif. Ce cas clinique démontre comment le LTNK peut avoir une présentation initiale aigue sous forme d'une cellulite orbitaire compliquant une pansinusite. L'absence d'amélioration sous traitement d'une cellulite orbitaire avec pansinusite doit alerter et faire suspecter un LTNK.

## Conflits d’intérêts

Les auteurs ne déclarent aucun conflit d'intérêts.
